# Urinary Test for Syphilis

**Published:** 1916-07

**Authors:** 


**Urinary Test for Syphilis.** Carl D. Gray, Portland, Me., Med. Rec., May 6. Fresh, acid urine, of normal s.g., above 1016, free from even a trace of sugar is used. To 6 C.C. of such urine, add 1 C.C. of a solution of resublimed iodine in chloroform or carbon tetrachlorid (strength not stated) shake thoroughly for several minutes and let stand. If the chloroform is pearly white, the test is negative. If pink or deep purple, it is positive, and syphilis is to be suspected. 1 C.C. of 10% aqueous solution of phosphoric acid is added in the latter case and the tube is again shaken and allowed to settle. If the color is now discharged, the test is negative. The author submits a report of 50 cases of syphilis in all of which the reaction was positive, as it was in all 5 of cases of nervous polyuria and all 10 of cases of polyuria produced by diuretics, and all 5 of cases of glycosuria, and in two cases of gonorrhoea and chancre, and in two of scarlet fever. Otherwise it was found positive in only one case, among 55 surgical cases. A table shows that the test usually corresponds with the results of the Wassermann. Urines of low s.g. always give a positive test. (Note: As stated by the author, the test evidently depends on the presence of an oxidizing agent (negative, colorless reaction) or on the absence of such a substance (colored, positive reaction). But, as recognized in the case of uric acid and diacetic acid, the amount of iodine must be definitely limited as a point is reached at which the oxidizing power is exhausted. We are inclined to think that this reaction cannot depend on any substance specific of syphilis but that the results merely indicate a general concurrence of certain metabolic states with syphilis or even that the drugs usually employed for syphilis produce the results).

